# Implementation of an efficient linear-optical quantum router

**DOI:** 10.1038/s41598-018-31273-0

**Published:** 2018-09-07

**Authors:** Karol Bartkiewicz, Antonín Černoch, Karel Lemr

**Affiliations:** 1 Faculty of Physics, Adam Mickiewicz University, PL-61-614 Poznań, Poland; 2 RCPTM, Joint Laboratory of Optics of Palacký University and Institute of Physics of Czech Academy of Sciences, 17. listopadu 12, 772 07 Olomouc, Czech Republic; 3 Institute of Physics of Czech Academy of Sciences, Joint Laboratory of Optics of Palacký University and Institute of Physics of Academy of Sciences of the Czech Republic, 17. listopadu 50A, 772 07 Olomouc, Czech Republic

**Keywords:** Quantum optics, Single photons and quantum effects

## Abstract

For several decades, scientists have been aware of significant benefits allowing quantum information processing technologies to surpass their classical counterparts. Recent technological development allows these benefits to be tested experimentally and in some cases also implemented in practical devices. So far the majority of experimental quantum networks was limited to peer-to-peer communications between two parties. Practical implementation of quantum communications networks, however, needs to address the problem of scalability to serve large numbers of users. Similarly to classical computer networks, their quantum counterparts would require routing protocols to direct the signal from its source to destination. Devices implementing these routing protocols are called quantum routers and have recently been subject of an intense research. In this paper, we report on experimental implementation of a linear-optical quantum router. Our device allows single-photon polarization-encoded qubits to be routed coherently into two spatial output modes depending on the state of two identical control qubits. The polarization qubit state of the routed photon is maintained during the routing operation. The success probability of our scheme can be increased up to 25% making it the most efficient linear-optical quantum router developed to this date.

## Introduction

**Figure 1:**
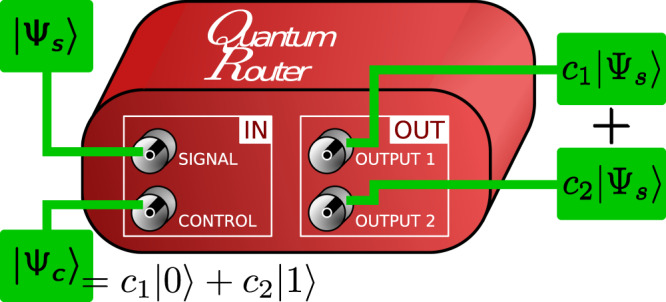
Conceptual scheme of a quantum router. Signal qubit is coherently routed into a superposition of output spatial modes with amplitudes given by the state of the control qubit.

Conceptual scheme of a quantum router^1^ is depicted in Fig. 1. The signal and control qubits denoted |*ψ*_*s*_〉 and |*ψ*_*c*_〉 = *c*_1_|0〉 + *c*_2_|1〉, respectively, serve as the router input. Based on the state of the control qubit, the signal is coherently forwarded to two output ports. Thus, the transformed signal state reads

1 $$|{\psi }_{s}\rangle \to {c}_{1}|{\psi }_{s}{\rangle }_{{\rm{OUT1}}}+{c}_{2}|{\psi }_{s}{\rangle }_{{\rm{OUT2}}}\,,$$

where indices OUT1 and OUT2 denote the two output ports. The quantum routing transformation belongs to a broader class of quantum state fusion protocols^2^ with the requirement to use spatially separate output ports. Note that in general quantum routers can operate on more than one signal and control qubits.

Quantum routers have been investigated both theoretically and experimentally for various experimental platforms^1,3–19^. Not all of these implementations can, however, be considered as fully quantum. In some cases, the routing information is classical and thus the router only semi-quantum^3,4^ in a sense that it classically routes a quantum state. Other implementations rely on non-linear interaction^5^ or combine various non-optical physical platforms making them impractical for realistic quantum networks^6–8^ due to ineffective and noisy interfaces. There are implementations that unavoidably disturb the inserted signal state and thus can not even be considered quantum routers at all^20^. While the cross-system interaction (e.g. light-atom interaction) introduces experimental challenges, the purely optical implementations face different shortcommings such as scalability issues or low success rates. A general quantum state fusion protocol implemented by Vitelli *et al*.^2^ meets all the requirements for a quantum router, but was not designed as such and operates with a rather low success probability of 1/8 (while applying feed-forward corrections).

In this paper, we report on an experimental implementation of a linear-optical quantum router based on our original theoretical proposal^1^. In contrast to the previous implementations, our device can reach success probability (routing efficiency) of up to 1/4. To our best knowledge, this makes it the most efficient quantum router on the platform of linear optics. Our device manages to reach this success probability by using of two identical copies (up to a constant phase shift) of the control qubit to route one signal qubit. Unless the control qubit is obtained from a computationally difficult operation, preparing two control qubits is not a serious obstacle to the practical usage of our routing protocol (e.g. prepration algorithm can run twice in parallel). Alternatively, using two different control qubits allows to generalize the scheme further. Such generalization is however beyond the scope of this paper. Note that the linear-optical implementation of quantum state fusion^2^ also utilizes three photons, where the third photon is used as an ancilla in a fixed input state.

## Router construction

**Figure 2:**
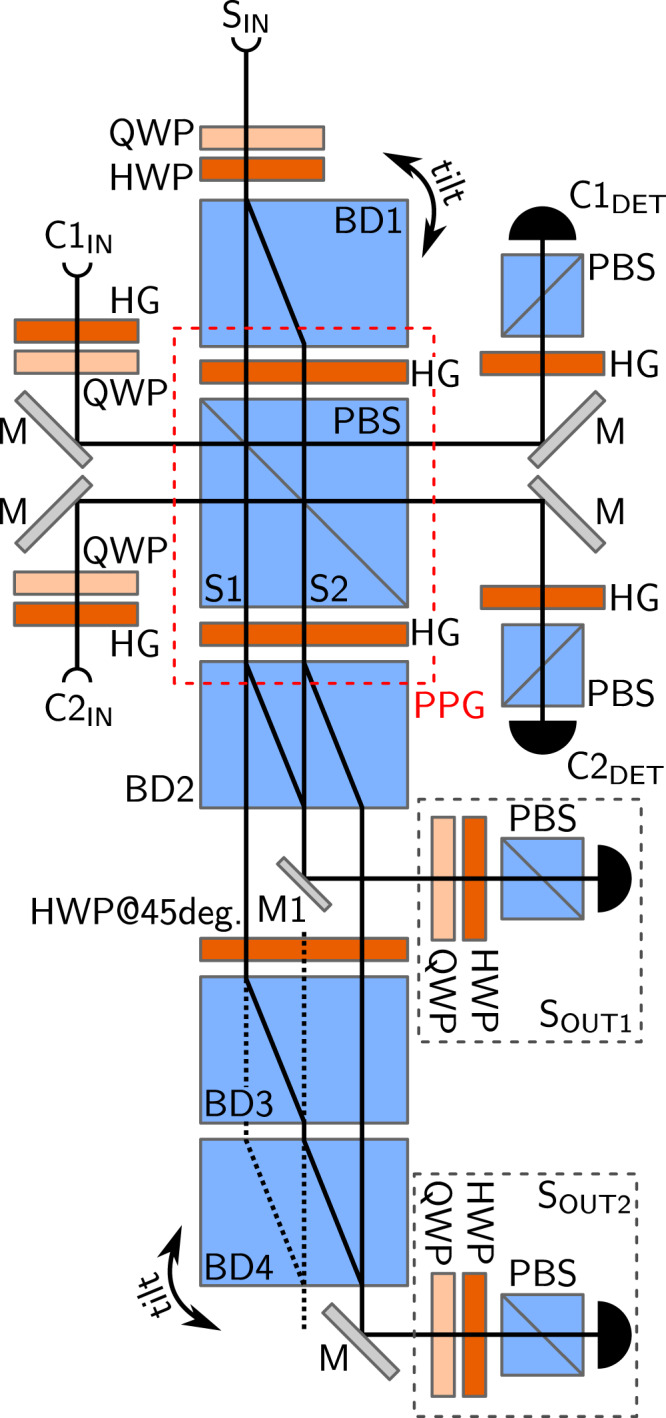
Experimental setup implementing the quantum router. Signal and control photons are inserted at S_IN_, C1_IN_ and C2_IN_, respectively. Control qubits are detected after being projected onto horizontal polarization states by detectors C1_DET_ and C2_DET_. Signal output state leaves the setup by two output ports denoted S_OUT1_ and S_OUT2_, where polarization analysis and detection of the signal takes place. Individual components are labelled as follows: PBS–polarizing beam splitter, BD–beam divider, HWP (QWP)–half- (quarter-)wave plate, HG–Hadamard gate (HWP rotated by 22.5 deg. with respect to horizontal polarization direction), M–mirror. Under normal operation, beams propagate along solid black lines. For coherence testing (as explained in the text), beams trajectories are changed to black dotted lines by removing mirror M1 and beam displacer BD3.

The working principle of our device can be understood by analyzing the experimental setup depicted in Fig. 2. The signal and control qubits are encoded in polarizations of single photons. Logical qubit states |0〉 and |1〉 are associated with the horizontal |*H*〉 and vertical |*V*〉 single-photon polarization respectively. The router is based on four beam dividers forming a complex but highly stable interferometer. This specific experimental construction has been selected because of its high interferometric stability. The signal qubit |*ψ*_*s*_〉 = *α*|*H*〉 + *β*|*V*〉 is prepared at the *S*_IN_ port by quarter and half wave plates. Next, it enters the first beam divider where its horizontal and vertical components are split into the spatial modes with the amplitudes *α* and *β*. Both these modes are subjected to a Hadamard gate implemented by a half-wave plate rotated by 22.5 deg. with respect to the horizontal polarization orientation. Subsequently, the two signal modes impinge on a polarizing beam splitter. On this beam splitter, each of these modes is coupled with one of the two control qubits that have been prepared in the state $|{\psi }_{c}\rangle =\frac{1}{\sqrt{2}}(|H\rangle +{{\rm{e}}}^{i\phi }|V\rangle )$. At the same time the state of the second control qubit is transformed, i.e., *φ* → *φ* + *π*. This can be achieved either by a HWP or as in our case directly during the state preparation. The parameter *φ* is the real-valued parameter defining the routing amplitudes in the output ports. It directly translates to the amplitudes *c*_1_ and *c*_2_ of the conceptual scheme by the relation $\phi ={\rm{atan}}|\frac{{c}_{1}}{{c}_{2}}|$. After interacting with the signal mode, each of the control qubits undergoes a Hadamard transform (using a half-wave plate) and is subsequently projected onto horizontal polarization state and detected. The block of half-wave plates and the polarizing beam splitter together implement the programmable phase gate (PPG) on each of the signal mode^21^. As a result, the signal modes acquire the phase shifts *φ* and *φ* + *π*. Thus, the signal photon state can be expressed in the form of

2 $$|{\psi }_{s}\rangle =\frac{\alpha }{\sqrt{2}}{(|H\rangle +{{\rm{e}}}^{i\phi }|V\rangle )}_{{\rm{S1}}}+\frac{\beta }{\sqrt{2}}{(|H\rangle -{{\rm{e}}}^{i\phi }|V\rangle )}_{{\rm{S2}}},$$

where indices S1 and S2 denote the spatial signal modes. These signal modes then are transformed by another Hadamard gate. In the final step, both these spatial signal modes are recombined on additional beam dividers. As a result the output signal state reads

3 $$|{\psi }_{s}{\rangle }_{{\rm{OUT}}}=\,\cos \,\tfrac{\phi }{2}{(\alpha |H\rangle +\beta |V\rangle )}_{{\rm{OUT1}}}-i\,\sin \,\tfrac{\phi }{2}{(\alpha |H\rangle +\beta |V\rangle )}_{{\rm{OUT2}}}.$$

The phase shift *π*/2 between the modes is insignificant and can be corrected by a phase shifter.

Projecting both the control qubits solely onto horizontal polarization makes both the included PPG gates operate with success probability of 1/4, thus, the router performs with the success probability of 1/16. One can immediately double the success probability by post-selecting also on projections onto vertical polarizations of the control qubits. If both of them are simultaneously projected onto vertical polarization, the router transformation remains identical but the output modes have to be classically swapped (e.g. using a classical optical switch). Yet another improvement in the success probability can be reached, if a feed-forward correction is implemented. As it was presented by Lemr *et al*.^10^, by means of a feed-forward correction, the PPGs can operate with success probability of 1/2. Thus, the router would reach the success rate of 1/4.

In the experiment, we post-select the successful router operation on three-fold coincidence detections. The two possible valid three-fold detections are the coincident detection in both control qubit output modes (detectors C1_DET_ and C2_DET_) together with either detection in the first signal output port S1_OUT_ or the second signal output port S2_OUT_. We refer to these three-fold coincident detections as CC1 and CC2, respectively. Typically, we observed about 1 three-fold coincidence per two minutes. Hence, it took several hours to accumulate hundreds of coincidences allowing to estimate the results with reasonably small uncertainties (assuming Poissonian distribution of the signal). To compensate for long-term power fluctuations, mainly due to the laser and coupling efficiency fluctuations, we swapped in two-minute intervals between two regimes during each measurement. In the first regime, we adjusted the mutual temporal delays between the three photons to be zero which made the photons interfere. In the second regime, the temporal overlap between the photons was deliberately detuned so that the photons did not interfere and the observed coincidence rate could be used for normalization.

## Results

Once the setup for the quantum router was constructed and adjusted, we performed a series of tests to verify that the device works properly, as described by Eq. (3). These tests were performed in three steps, each dedicated to verify one particular property of the operation. Technically, one can implement a complete process tomography to test all aspects of a quantum gate at once. Note however that quantum process tomography requires a large number of measurements (at least 256 different combinations of input states and output projections in this case). Considering the three-photon generation rate (1 per 2 minutes), such measurement would be here experimentally unfeasible.

**Figure 3:**
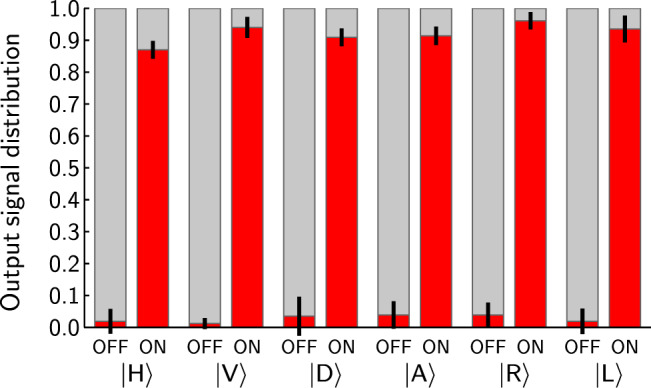
Probability of observing the signal photon leaving the router by the first (lightgrey upper portion of the bar) or the second (red lower segment of the bar) output port. The horizontal axis labels indicate the state of the control qubits (OFF and ON) and the state of the signal photon. Black segments centered at the top of each red bar depict the uncertainties of probability estimation. The presented probabilities are corrected by noise subtraction.

In the first step, we verified that depending on the phase shift *φ* the signal is routed to the first or the second output respectively. We denote the first control qubit state $|{\psi }_{c}\rangle =\frac{1}{\sqrt{2}}(|H\rangle +|V\rangle )$ (*φ* = 0) to be the logical state |0〉, i.e., the OFF state. Similarly, the first control qubit state $|{\psi }_{c}\rangle =\frac{1}{\sqrt{2}}(|H\rangle -|V\rangle )$ (*φ* = *π*) corresponds to the logical state |1〉, i.e., the ON state. Using this notation together with Eq. (3), one can easily determine that the signal leaves the device by the first output port, when the control qubits are in the OFF state. In contrary, the signal is routed exclusively into the second output port, when the control qubits read ON. For the purposes of this testing stage, we measured the rate of 3-fold coincidences CC1 and CC2 for both ON and OFF control states, and for the signal photon being in one of the six standard polarization states, i.e., horizontal |*H*〉, vertical |*V*〉, diagonal |*D*〉, anti-diagonal |*A*〉 linear polarization, and right-|*R*〉 and left-handed |*L*〉 circular polarization. In Fig. 3 we depict probabilities of observing the six states of the signal photon in the first and the second output port as a function of the control states OFF and ON (after subtracting the accidental coincidences from the total coincidence count, see Methods for more details). Our measurement certifies that the router directs the signal photon to the designated output port with a typical contrast above 20:1 (minimal corrected contrast was 11:1) based on the setting of the control qubits. Tabularized data as well as the raw data (before correcting for imperfect three-photon source) is presented in the Table 1. The first testing procedure verifies the capability of our device to route the signal correctly depending on the state of the control qubits.

**Table 1:** Probability *P*_2_ of observing the signal photon leaving the router by the second output port.

signal	control	*P*_2_	*σP*_2_	*P*_C2_	*σP*_C2_
|*H*〉	OFF	0.123	0.029	0.019	0.039
	ON	0.827	0.024	0.939	0.032
|*V*〉	OFF	0.145	0.011	0.012	0.017
	ON	0.840	0.025	0.940	0.033
|*D*〉	OFF	0.131	0.035	0.035	0.061
	ON	0.854	0.022	0.909	0.028
|*A*〉	OFF	0.174	0.029	0.039	0.043
	ON	0.855	0.023	0.914	0.029
|*R*〉	OFF	0.170	0.026	0.039	0.039
	ON	0.892	0.021	0.961	0.027
|*L*〉	OFF	0.141	0.028	0.019	0.040
	ON	0.825	0.026	0.935	0.042

Probability of observing the signal photon in first output is complement to unity, *P*_1_ = 1 − *P*_2_. *P*_C2_ denotes probability with correction on accidental coincidences.

**Figure 4:**
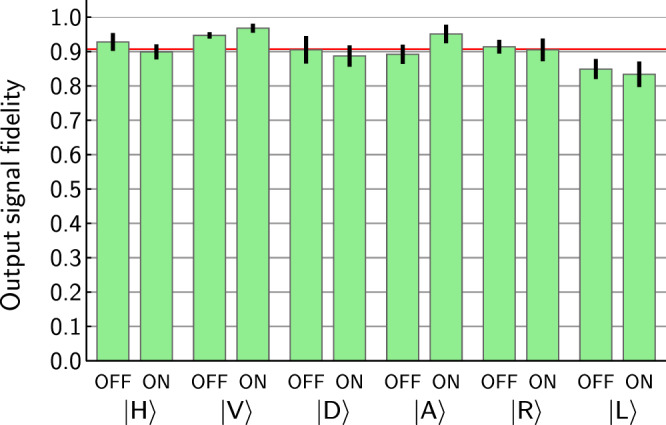
Output signal state fidelities measured for combinations of six input states and control qubit states OFF and ON. Heights of green bars correspond to fidelities, black segments centered at the top at each green bar mark the uncertainties of estimating the fidelities. Red line represents mean value 0.907. Presented fidelities are corrected by subtracting photon-source noise.

At the second stage we test if the signal state remains undisturbed by measuring output state fidelity for all the combinations of the six input signal states and two control states OFF and ON. The output state fidelity *F* = 〈*ψ*_*s*_|${\hat{\rho }}_{{\rm{sOUT}}}$|*ψ*_*s*_〉 indicates the overlap between the input state |*ψ*_*s*_〉 and in general mixed output signal state ${\hat{\rho }}_{{\rm{sOUT}}}$. Experimentally, fidelity *F* is obtained by projecting the output signal photons onto the input signal state and onto the orthogonal state. The ratio of these coincidence detection rates, denoted *R*, gives the fidelity $F=\frac{R}{1+R}$. Figure 4 shows the observed fidelities after subtracting the accidental coincidences from the directly measured rates. An average output state fidelity was found to be 0.907 ± 0.038. For observed values and raw data see Table 2. Causes for errors in the output signal state include imperfect two-photon bunching on PBS, imperfect single-photon interference and phase fluctuations in the interferometer. Based on the tests performed in the two above mentioned steps, we have estimated the process fidelity of the signal qubit to be 0.881 ± 0.034 and 0.847 ± 0.043 for the ON and OFF control qubit states respectively. At this point, we can certify that the router correctly redirects the signal photon and also quite reliably maintains its state.

**Table 2:** Output signal state fidelities measured for combinations of six input states and control qubit states OFF (fidelity measured on the first output) and ON (fidelity measured on the second output).

signal	control	*F*	*σF*	*F*_C_	*σF*_C_
|*H*〉	OFF	0.940	0.021	0.928	0.026
	ON	0.900	0.020	0.899	0.022
|*V*〉	OFF	0.959	0.007	0.947	0.009
	ON	0.972	0.011	0.968	0.013
|*D*〉	OFF	0.838	0.042	0.905	0.040
	ON	0.867	0.023	0.887	0.031
|*A*〉	OFF	0.871	0.021	0.892	0.028
	ON	0.883	0.022	0.951	0.027
|*R*〉	OFF	0.892	0.018	0.914	0.020
	ON	0.872	0.024	0.905	0.033
|*L*〉	OFF	0.805	0.024	0.849	0.029
	ON	0.778	0.028	0.834	0.037
mean		0.881	0.055	0.907	0.038

*F*_*C*_ denotes fidelity with correction on accidental coincidences.

**Figure 5:**
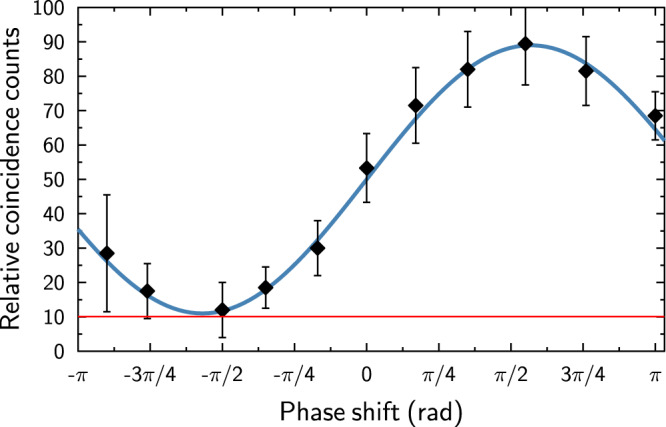
Relative coincidence counts CC2 measured for various phase shifts introduced by the tilt of beam displacer BD4. Black points represent the measured data, blue line is the theoretical fit by a harmonic function, and the red line shows the level of accidental coincidences as explained in the text.

The last test is to verify the capability of the router to route the signal photon coherently into a superposition of output ports. To investigate this aspect of the router, we have selected horizontally polarized input signal state |*ψ*_*s*_〉 = |*H*〉 and set the control qubits to $|{\psi }_{c}\rangle =\frac{1}{\sqrt{2}}(|H\rangle +i|V\rangle )$ which is a balanced superposition between the OFF and ON states. In this configuration, any possible decoherence effects would have the biggest impact on the observed visibility. To perform this test, the router setup was slightly modified. Namely the mirror M1 and beam displacer BD3 was removed. Note that this reconfiguration of the setup only serves the purpose of testing coherence between the output signal modes. The router normally operates on all possible signal and control states in the configuration explained in the router construction section. Projection onto diagonally polarized state was set in the output port S2_OUT_. In this modified setup, the signal photon is coherently routed by means of the PPG into two spatial modes which are subsequently overlapped on BD4 (see Fig. 2). By tilting this beam divider, we can introduce an arbitrary phase shift between the two interfering paths and thus observe interference fringes in detected coincidences CC2 behind a polarizer set to project onto diagonally polarized state. The coherence of the routing is thus translated into visibility of these interference fringes. We present our data in Fig. 5 demonstrating that once accidental coincidences are subtracted the visibility reaches 97.7% ± 0.3%. Visibility was calculated using the amplitude of a fitted harmonic function.

## Discussion

We have established that the router is able to send the signal state to the designated output port based on the state of the program qubits. The average output signal fidelity is well above the universal cloning threshold of 5/6 (see, e.g., work of Bartkiewicz and Miranowicz^22^ and the references therein), which guaranties that the input states are not copied individually and have the same origin^23^. We have also demonstrated the coherence of routing between the two output ports for a single input state. The coherence of routing should be maintained independently of the input state. In our setup this is ensured by the symmetry of the setup which maintains high fidelity of the output states. For the purposes of this proof-of-principle experiment, we operated the router in its basic regime with success probability of 1/16. This means that successful router operation was triggered by detecting control qubits in horizontal polarization states. Note that by adding a classical fiber switch the success rate can be improved to 1/8. In this case such classical switch would simply cross the output fibers when control qubits are both found to be vertically polarized. The probability of success can even reach 1/4 by applying feed-forward which implements the polarization transformation *V* → −*V* in the signal mode S1 and S2 when the associated control qubit is detected in vertical polarization state (see work of Miková *et al*.^24^). This represents a significant improvement in comparison with previously proposed similar devices.

## Methods

We use a femtosecond laser system Mira (Coherent) to generate pulses with repetition rate of 80 MHz, 800 mW mean power, central wavelength of 826 nm and spectral width of 10 nm (FWHM). These pulses are frequency doubled in the process of collinear second harmonics generation (SHG). Second harmonics is separated from the depleted fundamental beam by a dichroic mirror. Depleted fundamental spectral mode is then further attenuated by neutral density filter (NDF) to single-photon level (approximately 0.00125 photons per pulse) and serves as a source of signal photons for the router. The generated second harmonics with central wavelength of 413 nm is filtered spectrally by band-pass filter (10 nm FWHM) and spatially by 4 F system with an inserted pinhole. Remaining 100 mW of mean optical power pump the nonlinear BBO crystal and produces photon pairs in the Type-I process of spontaneous parametric down-conversion (SPDC). Approximate rate of obtained photon pairs is 2000 per second. All three optical modes–attenuated fundamental used as a signal (S_IN_) and down-conversion used as two controls (C1_IN_, C2_IN_)–are spectrally filtered by narrow-band filters with 3 nm in FWHM. Than the modes are coupled into single-mode optical fibers leading to three optical inputs of the main experimental setup–linear-optical quantum router.

Our three-photon source does not generate a perfect pure Fock |1〉 state at each of its outputs. The photon number statistics at the output ports is a product of SPDC and coherent state statistics. These imperfections cause the three-fold coincidences to be observed when, e.g., two photons were present at one of the inputs while another input was in a vacuum state. We call these instances accidental coincidence detections and their rate can be easily estimated using the theoretical framework developed by Trávníček *et al*.^25^. Knowing the typical single-photon detection rates from the SPDC process and from the attenuated fundamental beam, one can estimate the rate of accidental coincidences and subtract them from the overall detected coincidence rate. This procedure allows to describe the performance of the router independently on the imperfections of the source.

Typical rate of three fold coincidence counts (two controls and one of the signal outputs) was 1–2 per minute. This rate depends on the polarization projection on the signal outputs. To have low errors we have typically accumulated the data for 300 minutes for each setting of the router. Typical probability of accidental coincidences caused by multiple photons was 20%. Due to the polarization projection the effective rate of accidental coincidences was about ten times lower than the rate of coincidences originating from one photon at each input port.

More experimental details and tabularized values of the results are presented as the Supplementary information ^26^. The datasets generated during and analysed during the current study are available from the corresponding author on reasonable request.

## Electronic supplementary material

Supplementary information
