# Author Correction: Implementation of an efficient linear-optical quantum router

**DOI:** 10.1038/s41598-020-68524-y

**Published:** 2020-07-07

**Authors:** Karol Bartkiewicz, Antonín Černoch, Karel Lemr

**Affiliations:** 1 Faculty of Physics, Adam Mickiewicz University, 61-614 Poznań, Poland; 2 RCPTM, Joint Laboratory of Optics of Palacký University and Institute of Physics of Czech Academy of Sciences, 17. listopadu 12, 772 07 Olomouc, Czech Republic; 3 Institute of Physics of Czech Academy of Sciences, Joint Laboratory of Optics of Palacký University and Institute of Physics of Academy of Sciences of the Czech Republic, 17. listopadu 50A, 772 07 Olomouc, Czech Republic

Correction to: *Scientific Reports* 10.1038/s41598-018-31273-0, published online 07 September 2018

In Figure 3, the height of the second bar is incorrect and should be 0.939 as indicated in Table 1.

The correct Figure 3 appears below as Figure 1.

**Figure 1:**
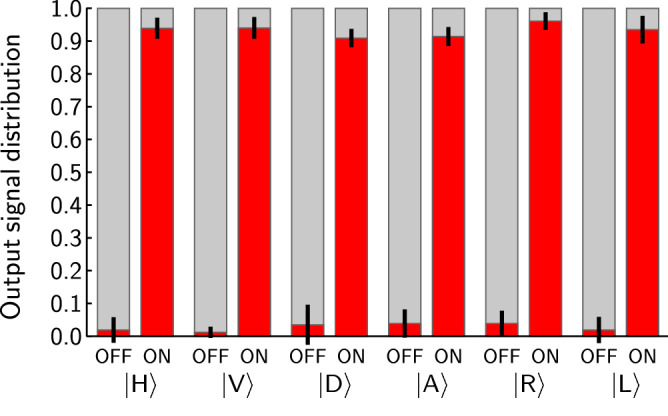
Probability of observing the signal photon leaving the router by the first (lightgrey upper portion of the bar) or the second (red lower segment of the bar) output port. The horizontal axis labels indicate the state of the control qubits (OFF and ON) and the state of the signal photon. Black segments centered at the top of each red bar depict the uncertainties of probability estimation. The presented probabilities are corrected by noise subtraction.

