# Supplementary material for: Implementation of an efficient linear-optical quantum router
Source: Sci Rep. 2018 Sep 7;8:13480. doi: 10.1038/s41598-018-31273-0 (PMC6128919; doi:10.1038/s41598-018-31273-0)
Supplement: Supplementary file 1 — Supplementary information [file 41598_2018_31273_MOESM1_ESM.pdf]

Implementation of an efficient linear-optical quantum router – Supplementary information

Karol Bartkiewicz,[1,2,*] Antonín Černoch,[3,†] and Karel Lemr[2,‡]

[1] *Faculty of Physics, Adam Mickiewicz University, PL-61-614 Poznań, Poland*
[2] *RCPTM, Joint Laboratory of Optics of Palacký University and Institute of Physics of Czech Academy of Sciences, 17. listopadu 12, 771 46 Olomouc, Czech Republic*
[3] *Institute of Physics of Czech Academy of Sciences, Joint Laboratory of Optics of PU and IP AS CR, 17. listopadu 50A, 772 07 Olomouc, Czech Republic*

(Dated: February 5, 2018)

I. THREE-PHOTON SOURCE

In this experiment, we used a typical three-photon source based on spontaneous parametric down-conversion (SPDC) and attenuated coherent state (see Fig. 1). We use femtosecond laser system Mira (Coherent) to generate pulses with repetition rate 80 MHz, 800 mW mean power, central wavelength 826 nm and spectral width 10 nm (FWHM). These pulses are frequency doubled in the process of collinear second harmonics generation (SHG). The upconverted light beam in separated on a dichroic mirror. The depleted fundamental mode is attenuated by neutral density filter (NDF) to single-photon level (approximately 0.00125 photons per pulse). The generated second harmonics with central wavelength of 413 nm is filtered spectrally by a pinhole in 4F system. Remaining 100 mW of mean optical power pump nonlinear crystal BBO to produce photon pairs in the Type-I process of spontaneous parametric down-conversion (SPDC). Approximate rate of photon pairs is 2 000 per second. All three optical modes – attenuated fundamental used as a signal (S_{IN}) and down-conversion used as two controls ($C1_{IN}$,$C2_{IN}$) – are spectrally filtered by narrow-band filters with 3 nm FWHM. Subsequently the modes are coupled into single-mode optical fibers leading to three optical inputs of the main experimental setup – linear-optical quantum router.

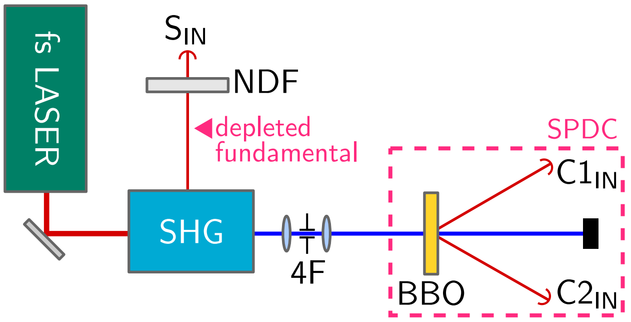

FIG. 1: Scheme of the three photon source. See description of components in the text.

The main problem of every three photon source of this type is noise caused by multiphoton contributions. In all three modes there is a nonzero probability of having generated two or more photons per laser pulse. And if the spatial modes are mixed on beam splitters as in our router than the multiphoton events can cause false three fold coincidence. These false coincidences have lower probability than the right ones but still they can affect the measurement results. Their rate can be easily found out by sequential blocking of each mode, see analysis in Ref. [1].

II. MEASUREMENT PROCEDURES AND OBTAINED DATA

Typical rate of three fold coincidence counts (two controls and one of the signal outputs) was 1–2 per minute. This rate depends on the polarization projection on the signal outputs. To have low errors we have typically accumulated the data for 300 minutes for each setting of the router. Typical probability of accidental coincidences caused by multiple photons was 20%. Due to the polarization projection the effective rate of noisy coincidences was ten times lower then right ones.

We present raw data without correction on false three fold coincidences and the corrected data for routing probabilities in the second output port in Table I. Uncorrected routing probabilities are visualized in Fig. 2. Mean contrast of the routing in first output mode without correction is $(5.7 \pm 0.9) : 1$, with correction then $(15.7 \pm 4.6) : 1$. In the second output port we obtain raw contrast $(5.8 \pm 0.6) : 1$ and corrected one $(41.8 \pm 19.7) : 1$.

In the table II, we show measured uncorrected and corrected output state fidelities on the first (control OFF) and the second (control ON) output port. The mean values for both cases are in the last row of the table. The uncorrected fidelities are also shown in Fig. 3.

The coherence between signal outputs OUT1 and OUT2 was tested without mirror M1 and beam displacer BD3 only for one input polarization. The relative phase

*Electronic address: bark@amu.edu.pl
†Electronic address: acernoch@fzu.cz
‡Electronic address: k.lemr@upol.cz

TABLE I: Probability P_2 of observing the signal photon leaving the router by the second output port. Probability of observing the signal photon in first output is complement to unity, $P_1 = 1 - P_2$. P_{C2} denotes probability with correction on accidental coincidences.

signal	control	P_2	σP_2	P_{C2}	σP_{C2}
$\vert H\rangle$	OFF	0.123	0.029	0.019	0.039
	ON	0.827	0.024	0.939	0.032
$\vert V\rangle$	OFF	0.145	0.011	0.012	0.017
	ON	0.840	0.025	0.940	0.033
$\vert D\rangle$	OFF	0.131	0.035	0.035	0.061
	ON	0.854	0.022	0.909	0.028
$\vert A\rangle$	OFF	0.174	0.029	0.039	0.043
	ON	0.855	0.023	0.914	0.029
$\vert R\rangle$	OFF	0.170	0.026	0.039	0.039
	ON	0.892	0.021	0.961	0.027
$\vert L\rangle$	OFF	0.141	0.028	0.019	0.040
	ON	0.825	0.026	0.935	0.042

TABLE II: Output signal state fidelities measured for combinations of six input states and control qubit states OFF (fidelity measured on the first output) and ON (fidelity measured on the second output). F_C denotes fidelity with correction on accidental coincidences.

signal	control	F	σF	F_C	σF_C
$\vert H\rangle$	OFF	0.940	0.021	0.928	0.026
	ON	0.900	0.020	0.899	0.022
$\vert V\rangle$	OFF	0.959	0.007	0.947	0.009
	ON	0.972	0.011	0.968	0.013
$\vert D\rangle$	OFF	0.838	0.042	0.905	0.040
	ON	0.867	0.023	0.887	0.031
$\vert A\rangle$	OFF	0.871	0.021	0.892	0.028
	ON	0.883	0.022	0.951	0.027
$\vert R\rangle$	OFF	0.892	0.018	0.914	0.020
	ON	0.872	0.024	0.905	0.033
$\vert L\rangle$	OFF	0.805	0.024	0.849	0.029
	ON	0.778	0.028	0.834	0.037
mean		0.881	0.055	0.907	0.038

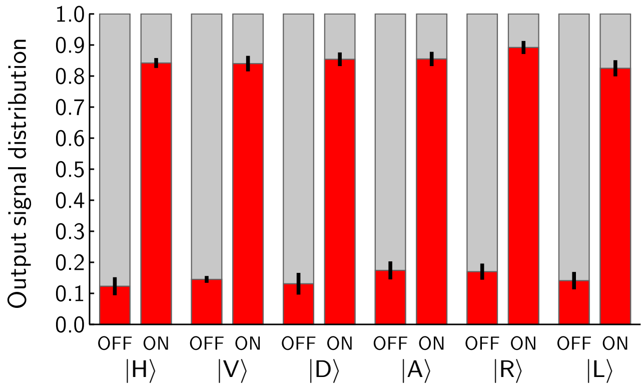

FIG. 2: Probability of observing the signal photon leaving the router by the first (lightgrey upper portion of the bar) or second (red lower segment of the bar) output port. The horizontal axis labels indicate the state of the control qubits (ON and OFF) and the state of the signal photon. Black segments centered at the top of each red bar depict the uncertainties of probability estimation. Presented probabilities are not corrected by noise subtraction.

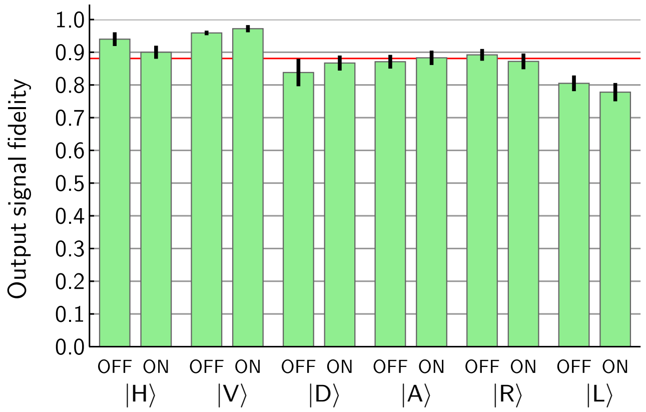

FIG. 3: Output signal state fidelities measured for combinations of six input states and control qubit states ON and OFF. Height of green bars correspond to fidelities, black segments centered at the top at each green bar mark the uncertainties of estimating the fidelities. Red line represents mean value 0.881. Presented fidelities are not corrected by noise subtraction.

TABLE III: Relative values of coincidence counts measured to test coherence between signal outputs.

phase [rad]	relative coincidences	error
-0.9	28.5	17
-0.76	17.5	8
-0.5	12.0	8
-0.35	18.5	6
-0.17	30.0	8
0.00	53.3	10
0.17	71.5	11
0.35	82.0	11
0.55	89.5	12
0.76	81.5	10
1.00	68.5	7

shift between different paths was tuned by the tilt of the last beam displacer BD4. For each phase we accumulated coincidence typically 50 minutes, see table Tab III. Due to the robust construction the phase in the interferometer remains stable for several hours. Without subtraction accidental coincidences we obtain visibility of interference fringe 76%. After correction on noise (with value 10.1) we obtain visibility $(97.7 \pm 0.3)\%$ (calculated from fitted sinus function).

[1] V. Trávníček, K. Bartkiewicz, A. Černoch, and K. Lemr, Phys. Rev. A **96**, 023847 (2017).
